# Use of whole genome expression analysis in the toxicity screening of nanoparticles

**DOI:** 10.1016/j.taap.2014.07.017

**Published:** 2014-10-15

**Authors:** Eleonore Fröhlich, Claudia Meindl, Karin Wagner, Gerd Leitinger, Eva Roblegg

**Affiliations:** a Center for Medical Research, Medical University of Graz, Stiftingtalstr. 24, 8010 Graz, Austria; b Institute for Cell Biology, Histology and Embryology, Medical University of Graz, Harrachgasse 21, 8010 Graz, Austria; c Institute of Pharmaceutical Sciences, Department of Pharmaceutical Technology, Karl-Franzens-University of Graz, Universitätsplatz 1, 8010 Graz, Austria

**Keywords:** Nanotoxicology, Whole genome expression analysis, Cytotoxicity, Oxidative stress, Interleukin secretion, Apoptosis

## Abstract

The use of nanoparticles (NPs) offers exciting new options in technical and medical applications provided they do not cause adverse cellular effects. Cellular effects of NPs depend on particle parameters and exposure conditions. In this study, whole genome expression arrays were employed to identify the influence of particle size, cytotoxicity, protein coating, and surface functionalization of polystyrene particles as model particles and for short carbon nanotubes (CNTs) as particles with potential interest in medical treatment. Another aim of the study was to find out whether screening by microarray would identify other or additional targets than commonly used cell-based assays for NP action. Whole genome expression analysis and assays for cell viability, interleukin secretion, oxidative stress, and apoptosis were employed. Similar to conventional assays, microarray data identified inflammation, oxidative stress, and apoptosis as affected by NP treatment. Application of lower particle doses and presence of protein decreased the total number of regulated genes but did not markedly influence the top regulated genes. Cellular effects of CNTs were small; only carboxyl-functionalized single-walled CNTs caused appreciable regulation of genes. It can be concluded that regulated functions correlated well with results in cell-based assays. Presence of protein mitigated cytotoxicity but did not cause a different pattern of regulated processes.

## Highlights

• Regulated functions were screened using whole genome expression assays.
• Polystyrene particles regulated more genes than short carbon nanotubes.
• Protein coating of polystyrene particles did not change regulation pattern.
• Functions regulated by microarray were confirmed by cell-based assay.

## Graphical abstract

**Figure:**
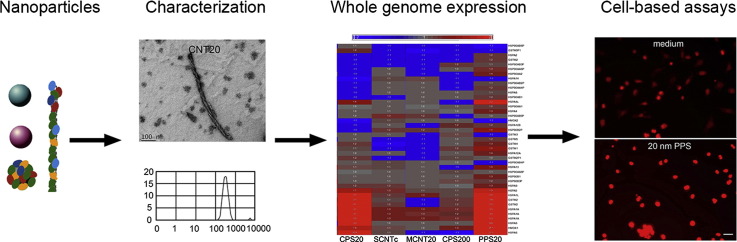

## Introduction

The use of nanoparticles (NPs) in consumer products and health products requires careful studies on their potential toxicological effects. Initial screening traditionally starts with assessment of viability by specific cell-based assays, based on the detection of cell number, enzyme activity, protein or ATP-content (Fröhlich et al., 2010). Advantages of these assays include ease of performance and interpretation and relative inexpensiveness. These assays, on the other hand, do not give information on the mode of toxicological action. This information is usually obtained after performance of additional assays, for instance for apoptosis, generation of reactive oxygen species or functional assays for organelle function.

Whole genome expression arrays have the advantage that they give information on a variety of cellular effects. They have been used to identify regulated pathways after exposure to NPs in cells and tissues (Coccini et al., 2012, Gui et al., 2013, Waters et al., 2009). Since they can provide an overview on the changes occurring under different exposure conditions they are useful for identifying the role of specific NP parameters on gene regulation.

Studies in RAW264 macrophages showed that particle size had a prominent effect on gene expression. While cell cycle, apoptosis, cell differentiation and signal transduction were more regulated by large (500 nm) particles, small (10 nm) silica (SiO_2_) particles had a stronger effect on morphogenesis (Waters et al., 2009).

It has also been suggested that highly cytotoxic particles induce another gene expression profile than less cytotoxic particles (Tilton et al., 2014). Cytotoxicity of an NP on a given cell depends on its concentration and on the composition of the medium in which the NP was applied. In order not to change physicochemical parameters of NPs and cause unwanted aggregation, many studies exposed cells to NPs suspended in protein-free solution (saline, buffer, cell culture medium without protein, etc.). When tested in the presence of serum, some types of particles, for instance polystyrene particles, act less cytotoxic than in the absence of protein (Fröhlich et al., 2009). It is not clear whether this is due to decreased interaction of particle surface with the plasma membrane or to induction of different cellular mechanisms.

NPs consisting of different materials, like titanium dioxide (TiO_2_) and zinc oxide (ZnO) NPs, affected similar cell functions, such as apoptosis, reactive oxygen generation, and inflammation (Moos et al., 2011). Since it has been postulated that immunological effects of particles are mainly determined by their surface properties, the reason for the similarity between different particles could be the fact that they possessed similar coating with macromolecules. The binding of macromolecules, mostly proteins, generally termed ‘protein corona,’ plays a predominant role for particle size and cytotoxicity. Composition and concentration of the proteins mainly determine the composition of the coating (Ghavami et al., 2013, Monopoli et al., 2011, Tenzer et al., 2013).

In our study we aimed to address the effect of particle parameters and protein coating on gene regulation using carboxyl and plain polystyrene particles applied in different media and at different concentrations. In addition, short CNTs as examples for medically relevant NPs were tested. In contrast to the long tubes, short CNTs present a more favorable toxicological profile and could be suitable for application in gene therapy, immunotherapy, tissue regeneration, medical imaging and chemotherapy (He et al., 2013). Since potential medical applications for CNTs are preferentially parenteral, endothelial cells were used for the screening. EAhy926 endothelial cells, a fusion of human umbilical vein endothelial cells with the lung adenocarcinoma cell line A549, were shown to express endothelial-specific markers such as endothelin-1, prostacyclin, VIII-related antigen (von Willebrand factor), endothelial adhesion molecules ICAM-1 and VCAM-1 (Edgell et al., 1983, Edgell et al., 1990, Emeis and Edgell, 1988, Saijonmaa et al., 1991, Suggs et al., 1986, Thornhill et al., 1993, van Oost et al., 1986). This expression pattern demonstrates well-preserved differentiation as endothelial cells. EAhy926 cells are the most studied and characterized permanent human endothelial cell line (Bouis et al., 2001) and have also been reported to be a preferable homogeneous experimental model with greater reproducibility of data than human umbilical vein endothelial cells (Eremeeva and Silverman, 1998).

To address the role of size, NPs with different diameters were compared. Testing of NPs in different concentrations and of NPs in different media was used to study the role of cytotoxicity. To investigate the role of protein coating NPs suspended in cell culture medium with different fetal bovine serum (FBS) content and exposure to different NPs suspended in the same medium were used. The influence of surface charge was studied by comparison of carboxyl-functionalized and plain NPs. Genes that responded to NPs (differentially expressed genes) will be termed ‘regulated genes.’ Some of the regulated genes (interleukin 6 and interleukin 8) and functions (apoptosis, and oxidative stress) identified by whole genome expression profiles were validated by cellular assays.

## Materials and methods

### Particles

20 and 200 nm carboxyl polystyrene particles (Invitrogen), 20 and 200 nm plain polystyrene particles (Thermo Scientific) and short (0.5–2 μm) carbon nanotubes (Cheap Tubes) as single-walled tubes (SCNTs, 1–2 nm) and as multi-walled tubes (MCNTs) in diameters of < 8 nm, 20–30 nm, and > 50 nm were used. Particles were diluted with the respective medium and sonicated in an Elmasonic S40 water bath (ultrasonic frequency: 37 kHz, 40 W, Elma, Singen) for 20 min.

### Physico-chemical characterization

NPs were characterized immediately after ultrasound treatment and all dilutions were prepared from a freshly prepared stem solution of 1 mg/ml particles in the respective medium.

#### Characterization by dynamic light scattering

Particles were characterized regarding size and zeta potential was measured by dynamic light scattering and laser doppler velocimetry using a ZetaSizer Nano-ZS (Malvern Instruments, UK). Polystyrene particles were characterized at a concentration of 200 μg/ml, CNTs at a concentration of 1 μg/ml. After equilibration of the sample solution to 25 °C, size and zeta potential were measured at 633 nm and a detection angle of 90°. NNLS software was used for sample analysis.

#### Characterization by transmission electron microscopy

The CNTs were dispersed in DMEM cell culture medium at 1 mg/ml dilution and treated with ultrasound for 20 min. 5 μl of this solution was placed on a carbon coated copper grid that had previously been treated with a PELCO easiGlow Glow Discharge device. After 1 min incubation, the solution was withdrawn using non hardened microscopic filter paper (Whatman).

Images were taken using a FEI Tecnai G^2^ 20 transmission electron microscope with a Gatan ultrascan 1000 ccd camera. Acceleration voltage was 80 kV. Sizes of CNTs were measured from the TEM images.

For energy dispersive X-ray spectroscopy (EDX), the specimens were diluted in double distilled water, sonicated, and placed on pioloform-coated nickel grids. Scanning transmission EM (STEM) micrographs were made with an FEI Tecnai 20 electron microscope at 20,000 × magnification and 120 kV acceleration voltage with a high angle annular dark field detector. EDX spectra were made with a Standard SUTW detector (EDAX) in rectangular selections within the field of view of these micrographs and each spectrum was recorded for 2 min. Data on the elemental composition were gained using Peak ID and Quantify functions of TEM imaging and analysis (TIA) software, FEI company.

### Cell culture

The human endothelial cell line EAhy926 (kind gift from Dr. C. J. Edgell) was cultured in DMEM, 10% FBS, 2 mM l-glutamine and 1% penicillin/streptomycin. Cells were cultured at 37 ° C in a humid 95% air/5% CO_2_ atmosphere. Cells were pre-cultured in this medium 24 h before treatment.

### Cellular uptake

Internally red fluorescently labeled 20 nm and 200 nm plain polystrene particles (FluoroMax red, ThermoScientific) and red fluorescently labeled carboxyl polystyrene particles (FluoSpheres, Invitrogen) were used. CNT uptake was assessed by labeling of the CNTs with fluorescent bovine serum albumine (BSA) because absorption measurements of unlabeled black CNTs was too insensitive to allow quantification of cellular uptake. Labeling and quantification of CNT uptake was performed according to the protocol by Mu et al. (2009). Briefly, 1 mg/ml deionized water BSA Alexa Fluor 555 conjugate (Invitrogen) was added to 1 mg/ml CNTs suspended in water and incubated for overnight at 4 °C. To remove un-bound BSA, CNTs were centifugated at 16,000*g* for 30 min, re-suspended and centrifuged again five times. CNTs were labeled the day prior to the experiments. When labeled CNTs were stored for 7 days and centrifuged again, significant amounts of fluorescence was detected in the supernatant, indicating stability problems of the labeled CNTs. This effect was not seen when labeled CNTs were centrifuged after 3 days.

Cells were incubated with 20 μg/ml of the fluorescent polystyrene particles and 50 μg/ml CNTs for 24 h at 37 °C. Thereafter, cells were detached from the plastic support treatment with 0.05% trypsin/EDTA, suspended in exposure medium and washed by repeated centrifugation at 800 rpm for 5 min. Fluorescence was read at Ex/Em wavelength of 584/612 nm (FluoSpheres), 544/612 nm (FluoroMAx), and 544/590 nm (fluorescent- labeled CNTs) using a fluorescence plate reader (FLUOstar Optima, BMG Labortechnik). Dilutions of the stock solutions were prepared with cell homogenates to account for interference of the cells with the fluorescent signal. Uptake was normalized to fluorescence of the stock solutions as 100%. In parallel cellular uptake was visualized by microscopy. Images were taken at a LSM510 Meta (Zeiss).

### Cellular localization

For intracellular localization internally green fluorecently labeled 20 nm and 200 nm carboxyl polystyrene particles (YG Fluospheres, Invitrogen), plain polystyrene particles (FluoroMax green, ThermoScientific) and CNTs after labeling with BSA- 488 Alexa (Invitrogen) (labeling see Cellular uptake section) were used. For labeling of mitochondria cells, after the incubation with NPs or medium, were rinsed twice in medium + 10% FBS and incubated in 200 nM MitoTracker CMXRos in complete medium for 20 min at 37 °C. After washing with complete medium cells nuclei were stained by incubation with Hoechst33342 (1 μg/ml) for 15 min at RT. Confocal images were taken at a LSM510 Meta (Zeiss).

### Cytotoxicity screening (formazan bioreduction)

CellTiter 96® AQueous Non-Radioactive Cell Proliferation Assay (Promega) was used according to the manufacturer's instructions. For adherent cells CNTs were removed by repeated washing with PBS. 20 μl of the combined MTS/PMS solution was added to 100 μl of each well and plates were incubated for 2 h at 37 °C in the cell incubator. The supernatant after formation of the formazan product was transferred to a new plate to ensure that the signal was not influenced by absorbance of CNTs incorporated into cells. Absorbance was read at 490 nm on a plate reader (SPECTRA MAX plus 384, Molecular Devices). For PPS and CPS absorbance was read in the same plate and the absorbance of the particles without cells was substracted from the signal.

### Microarray experiments

Whole genome microarray analysis was performed using GeneChip® Human Gene 1.0 ST Array (Affymetrix) for protein coding and long intergenic non-coding RNA transcripts. EAhy926 cells (300.00 cells/6-well plate, pre-cultured for 48 h) were exposed to 150 μg/ml and 200 μg/ml CPS20 and CPS200 particles and 10 μg/ml PPS particles were suspended in DMEM for 6 h, and to 200 μg/ml PPS particles, 20 μg/ml and 50 μg/ml CNT in DMEM + 10% FBS for 24 h. At these doses minimal cytotoxicity was observed. Shorter time points were not investigated because it is known that after short exposure times, such as 1 h, cell-specific but not NP-specific responses are obtained (Tilton et al., 2014).

### Hybridization

Total RNA was isolated using RNeasy Mini kit (Qiagen) according to the manufacturer's recommendations. The integrity of each RNA sample was evaluated using an Agilent 2100 Bioanalyzer (Agilent) and only RNAs with an RNA integrity number (RIN) above 9.1 were used for hybridizations. 100 ng of total RNA for each sample were processed using the Affymetrix GeneChip Whole Transcript (WT) Sense Target Labeling Assay according to the manufacturer's instructions (Affymetrix). Double stranded cDNA was synthesized using a random hexamers tagged with a T7 promoter sequence. Using in vitro transcription, cRNA was generated from the double-stranded cDNA template using the Whole Transcript cDNA Synthesis and Amplification Kit (Affymetrix). cDNA was regenerated using a reverse transcription reaction randomly primed with a mix containing dUTP. After hydrolysis of the cRNA with RNase H, the sense strand of cDNA was purified using the Affymetrix sample cleanup module, fragmented by incubation with UDG (uracil DNA glycosylase) and APE 1 (apurinic/apyrimidic endonuclease 1), and terminally biotin-labeled with terminal deoxynucleotidyl transferase using the WT Terminal Labeling Kit (Affymetrix), following the manufacturer's instructions. Biotinylated sense strands were fragmented and hybridized to Affymetrix Human GeneChip 1.0 ST arrays (Affymetrix) using the Hybridization Control and Hybridization Wash and Stain kits (Affymetrix). The hybridization cocktail was incubated overnight at 45 °C while rotating in a hybridization oven. After 16 h of hybridization, arrays were washed and stained in an Affymetrix GeneChip fluidics station 450, according to the Affymetrix-recommended protocol. Arrays were scanned on an Affymetrix GeneChip scanner GCS3000.

### Data analysis

CEL files were imported into Partek Genomic Suite 6.6 software (Partek Inc., St. Louis, MO) and robust multi-chip average (RMA) normalized (including background correction, quantile normalization across all arrays, median polished summerization based on log transformed expression values). For detection of differentially expressed genes analysis of variance (ANOVA) was performed and genes with p < 0.05 and a fold change of at least 1.5 were considered to be significantly regulated. To identify functional pathways out of a set of differentially express genes Ingenuity IPA (interactive pathway analysis; 18-9-2013: version: 16542223 buit: ing_xiphias; http://www.ingenuity.com) was used.

### Interleukin secretion (ELISA)

Supernatants of the cells exposed to NPs for 6h and for 24h and medium without particles were assessed. The release of IL-6 and IL-8 was measured using the human IL-6 ELISA set (BD OptEIA™) and the human IL-8 ELISA set (BD OptEIA™, BD Biosciences) according to the protocol given by the producers. Absorbance was read at 450 nm on a SPECTRA MAX plus 384 photometer (BMG Labortechnik).

### Oxidative stress (oxidation of dehydroethidium)

Generation of radicals was evaluated after 6 h and after 24 h in the presence of 10 μM DHE (Invitrogen) and particles or 200 μM H_2_O_2_ in cell culture medium. Cultures exposed to H_2_O_2_ and to polystyrene particles were analyzed immediately, while cells exposed to CNTs were rinsed three times in PBS. Fluorescence was read with 544 nm excitation and 612 nm emission at a FLUOstar Optima (BMG Labortechnik). For verification of the results, cells were also viewed under a LSM510 Meta confocal microscope.

### Apoptosis (YoPro-1 staining)

EAhy926 cells were treated with NPs suspended in the respective medium for 6 h and 24 h. Cultures exposed to polystyrene particles were analyzed immediately, while cells exposed to CNTs were rinsed three times in PBS. 100 μl of the stock solution (Invitrogen) was added to 1 ml DMEM, mixed and incubated for 30 min at 4 °C in the dark. Fluorescence was read at Ex/Em wavelength of 485/520 nm using a fluorescence plate reader (FLUOstar Optima) and cells were viewed with LSM510 Meta confocal microscope YoPro-1 signals after incubation with 100 μM H_2_O_2_ served as positive control.

### Statistics

These data are represented as means ± S.D. Data has been analyzed with one-way analysis of variance (ANOVA), followed by a Tukey-HSD post hoc test for multiple comparisons (SPSS 19 software).

## Results

Various exposure conditions were used to study the role of size, cytotoxicity, protein coating, and surface functionalization of NPs on gene regulation of endothelial cells. A summary of these exposures is provided in Table 1.

**Table 1:** List of exposure conditions.

Parameter	Exposures used for comparisons
Size/diameter (primary particle)	CPS20, CPS200 (200 μg/ml) in 0% FBS, PPS20 (200 μg/ml), SCNTc, MCNT20 (50 μg/ml) in 10% FBS
Biological effect (protein coating)	PPS20 (10 μg/ml) in 0% FBS; PPS20 (200 μg/ml) in 10% FBS
Medium (particle changes)	CPS20 (200 μg/ml) in 0%, 1%, 10% FBS
Dose (different cytotoxicity)	CPS20 (150 μg/ml, 200 μg/ml) in 0% FBS; SCNTc, MCNT20 (20 μg/ml and 50 μg/ml) in 10% FBS
Surface (carboxylation)	CPS20 (200 μg/ml), PPS20 (10 μg/ml) in 0% FBS; all CNTs (20 μg/ml, 50 μg/ml) in 10% FBS

### Physico-chemical properties

Various commonly particle characterization methods have to be employed to get information on diameter, chemical composition, and surface charge of the NPs (Hassellov et al., 2008). Diameter was determined by dynamic light scattering and transmission electron microscopy. While light scattering determined the hydrodynamic diameter of spherical particles, transmission electron microscopy was used to measure length and diameter of CNTs. Laser doppler velocimetry served to indicate zeta potential, which is determined by components adsorbed to the particle surface and provides information on the stability of the solution and the effectiveness of the surface charge in the solution (Müller, 1996). The composition of the NPs was determined by energy dispersive X-ray spectroscopy.

In medium (DMEM) without protein (FBS), 20 nm PPS and CPS particles were smaller and zeta potential of CPS more negative than in medium with protein; size of 20 nm CPS particles was larger and zeta potential more negative compared to 20 nm PPS particles (Table 2a). Since CNTs could only be dispersed in DMEM + 10% FBS and not without protein, CNTs were determined in this medium by transmission electron microscopy (TEM) and by dynamic light scattering and laser doppler velocimetry. As reported previously, zeta potential for both types of CNTs, carboxyl-functionalized and pristine CNTs, was slightly negative, indicating that all particles possessed a tendency to agregate in the suspension solution (Table 2b, Fröhlich et al., 2013). CNT sizes by TEM showed no significant deviations from the nominal diameters for most CNTs. Only SCNTs were seen exclusively in bundles and thickness of single tubes could not be reported exactly; MCNT8c were also in part aranged in bundles, while all other CNTs did not form bundles but aggregates. Mean length of the different non-aggregated CNTs ranged between 217 nm and 446 nm. Bundles of CNTs, formed by SCNT, SCNTc, and MCNT8c were between 543 nm and 816 nm long. Most aggregates, seen in bright field microscopy, were between 5 and 10 μm but aggregates up to 100 μm (data not shown) also occurred.

**Table 2a:** Characterization of polystyrene particles in different media by PCS.

Particle	Size (nm)		Zeta potential (mV)	
	DMEM	DMEM + 10% FBS	DMEM	DMEM + 10% FBS
PPS 20 nm	29	74	− 13	− 12
PPS 200 nm	212	224	− 11	− 8
CPS 20 nm	42	80	− 37	− 8
CPS 200 nm	230	212	− 42	− 10

**Table 2b:** Characterization of carbon nanotubes suspended in DMEM + 10% FBS by PCS and TEM.

	DLS/DLV data		TEM data			
Carbon nanotube	Hydro-dynamic size (nm)	ζ (mV)	Diameter of single CNTs (nm)	Length of single CNTs (nm)	Diameter of CNT bundles (nm)	Length of CNT bundles (nm)
SCNT	16	− 10	~ 2 nm	n.d.	28.3 ± 10.6	543 ± 60.8
SCNTc	15	− 8	~ 2 nm	n.d.	62.5 ± 41.9	816 ± 275.4
MCNT8	26	− 7	4.7 ± 0.48	222 ± 126.2	n.a.	n.a.
MCNT8c	16	− 10	4.2 ± 0.8	217 ± 117.9	24.3 ± 5.1	600 ± 282.8*
MCNT20	124	− 10	18.9 ± 0.9	446 ± 77.9	n.a.	n.a.
MCNT20c	38	− 10	15.3 ± 2.5	251 ± 94.4	n.a.	n.a.
MCNT50	52	− 7	62.8 ± 5.7	355 ± 96.4	n.a.	n.a.
MCNT50c	50	− 11	63.6 ± 11.3	392 ± 195.3	n.a.	n.a.

**Fig. 1:**
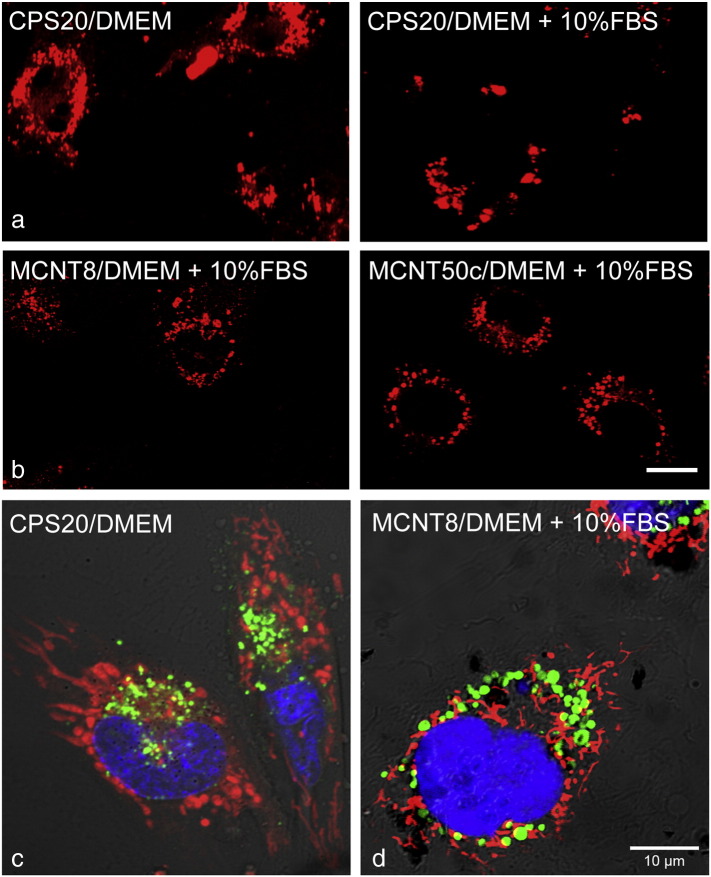
Uptake of fluorescently labeled NPs into EAhy926 cells. Images showing samples used for the quantification of uptake by fluorometry. Cells show uptake of red fluorescently labeled CPS20 particles suspended in DMEM without FBS and in DMEM + 10% FBS (a) and of red fluorescently labeled MCNT8 and MCNT50c suspended in DMEM + 10% FBS (b). Scale bar: 20 μm. For co-localization studies, green fluorescently labeled NPs were used to allow discrimination of particles and mitochondria identified by the red fluorescent dye MitoTracker CMXRos. Localization of CPS20 (c) and MCNT8 particles (d) is shown. Nuclei were counterstained with Hoechst33342 (blue).

To identify metal contaminants from residues of catalysts, X-ray energy dispersive spectrum data were obtained of the CNTs. In addition to carbon, only O, Si and Ni were detected. The presence of Si in the samples can either be explaind by an internal fluorescence peak stemming from the silicium detector or by the dispersion of CNTs in water, where Si had dissolved from glass bottles. Ni originates from the grid on which the CNTs were viewed (Fig. 1s, Supplementary Material).

### Cellular uptake and cytotoxicity of NPs

EAhy926 cells ingested fluorescently labeled 20 nm and 200 nm CPS and PPS particles after 6 h. For all CPS and PPS uptake in DMEM + 10% FBS was lower than in DMEM without FBS (Fig. 1a). FBS-dependent differences in particle uptake were slightly more pronounced for CPS than for PPS particles. The influence of FBS was also greater for 20 nm than for 200 nm polystyrene particles. Uptake of thin and thick CNTs was in the same order of magnitude (Fig. 1b). Ingested NPs, MCNT8 and CPS20 particles shown as examples, were localized between mitochondria (Fig. 1c,d). Fluorescence readings showed the same trend (Table 3).

**Table 3:** Particle uptake (% of applied) after exposure of EAhy926 cells to fluorescently labeled NPs (20 µg/ml polystyrene particles and 50 µg/ml CNTs) under the conditions used for the whole genome analysis studies.

Medium	Particles					
DMEM +	PPS20	PPS200	CPS20	CPS200	CNTs ≤ 8 nm	CNTs ≥ 20 nm
0% FBS	3.1 ± 0.9	8.6 ± 0.4	2.9 ± 0.3	10.3 ± 0.6	n.a.	n.a.
1% FBS	n.a.	n.a.	1.4 ± 0.2	7.1 ± 0.3	n.a	n.a
10% FBS	2.0 ± 0.9	2.4 ± 0.4	1.5 ± 0.2	1.7 ± 0.1	1.3 ± 0.2 to 8.1 ± 0.3	2.1 ± 0.6 to 8.9 ± 2.4

Cytotoxicity testing was performed to determine the concentration causing minimal cytotoxicity and to discriminate between high and low cytotoxic NPs. Expression analysis were intended to be performed close to cytotoxic concentration for both polystyrene NPs and CNTs under the assumption that these concentrations were the most likely to cause cellular effects. For CNTs a concentration of 50 μg/ml is not totally unrealistic because NPs for delivery of daunorubicin (peak plasma levels of 33.4–52.3 μg/ml) liposome concentrations are 585–917 μg/ml (Feingold et al., 2010).

**Fig. 2:**
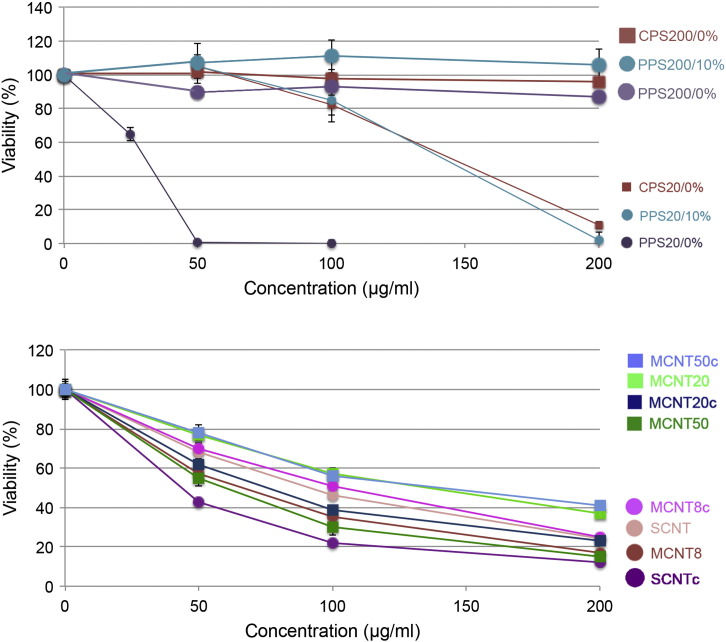
Viability of EAhy926 cells assessed by formazan bioreduction (dehydrogenase activity) in the presence of NPs for 24 h. a: Small polystyrene particles (small dots) show stronger cytotoxicity than larger particles (larger dots). b: Thin CNTs (red and pink curves) show higher cytotoxicity than thick CNTs (green and blue curves). Viability is indicated related to the dehydrogenase activity of cells in the respective medium as 100%.

Exposure to 200 μg/ml PPS20 in DMEM + 10% FBS and to 200 μg/ml CPS20 particles in DMEM caused significant cytotoxicity after 24 h (Fig. 2a). After 4 h, 200 μg/ml PPS20 and 200 μg/ml CPS20 particles did not reduce viability significantly (data not shown). PPS20 particles suspended in DMEM without FBS reacted much more cytotoxic and 10 μg/ml PPS20 caused the same decrease in viability as 200 μg/ml PPS20 particles suspended in DMEM + 10% FBS. When CPS20 particles were assessed in DMEM + 1% FBS for 24 h, cytotoxicity was less pronounced than at the same concentration of CPS20 particles in DMEM without FBS; instead of 5 ± 0.5% viability at 200 μg/ml CPS20 particles after exposure to DMEM without FBS, viability was 68 ± 9% (data not shown). At 10% FBS in the medium no cytotoxicity was observed up to 1 mg/ml CPS20 particles (data not shown). 50 μg/ml SCNTc, being the most cytotoxic of the CNTs tested, caused no significant decrease in viability (Fig. 2b). To verify absence of cytotoxicity in the microarray experiments viability testing was performed in parallel (data not shown).

### Whole genome expression analysis

To describe expression changes upon NP exposure two ways of analysis were used. In the analysis for regulated genes the 20 highest up-regulated and the 10 highest down-regulated genes were listed and classified according to their main function. Categories were inflammatory processes, DNA damage, oxidative stress, apoptosis, cell cycle, tyrosine kinase signaling, mitochondrial metabolism, endothelial specific genes and proteases. For classification information available at GeneCards® (http://www.genecards.org/) was used. For this analysis genes were filtered for fold changes of ≥ 1.5, p < 0.05 and correction for multiple comparisons (FDR) of 5%.

In the analysis for regulated functions well-annotated genes with fold changes of ≥ 1.5 and p < 0.05 were included. Functions were analyzed using ingenuity pathway analysis (IPA), which needs a minimum number of regulated genes. Therefore, the FDR of 5% was not applied.

Depending on the question, like role on size, cytotoxicity, protein coating, and surface functionalization, the respective data sets were compared.

In total, the number of regulated genes was much higher for polystyrene particles than for CNTs (Table 4).

**Table 4:** Number of well-annotated differentially regulated genes in NPs exposed cells compared to the respective medium exposed controls.

Particles	Number of well annotated differentially regulated genes		
	All genes	Up-regulated	Down-regulated
PPS20, DMEM	229	161	68
PPS20, 10% FBS	1775	891	884
CPS20, 150 μg, DMEM	659	378	281
CPS20, DMEM	1257	653	604
CPS200, DMEM	32	12	20
CPS20, 1% FBS	384	326	58
CPS200, 1% FBS	128	59	69
CPS20, 10% FBS	9	5	4
CPS200, 10% FBS	16	8	8
SCNT, 20 μg/ml	31	12	21
SCNTc, 20 μg/ml	23	14	9
SCNTc, 50 μg/ml	86	40	46
MCNT8, 20 μg/ml	45	15	30
MCNT8c, 20 μg/ml	17	4	15
MCNT20, 20 μg/ml	3	1	2
MCNT20, 50 μg/ml	4	2	2
MCNT20c, 20 μg/ml	6	5	4
MCNT50, 20 μg/ml	6	5	1
MCNT50c, 20 μg/ml	6	2	4

**Fig. 3:**
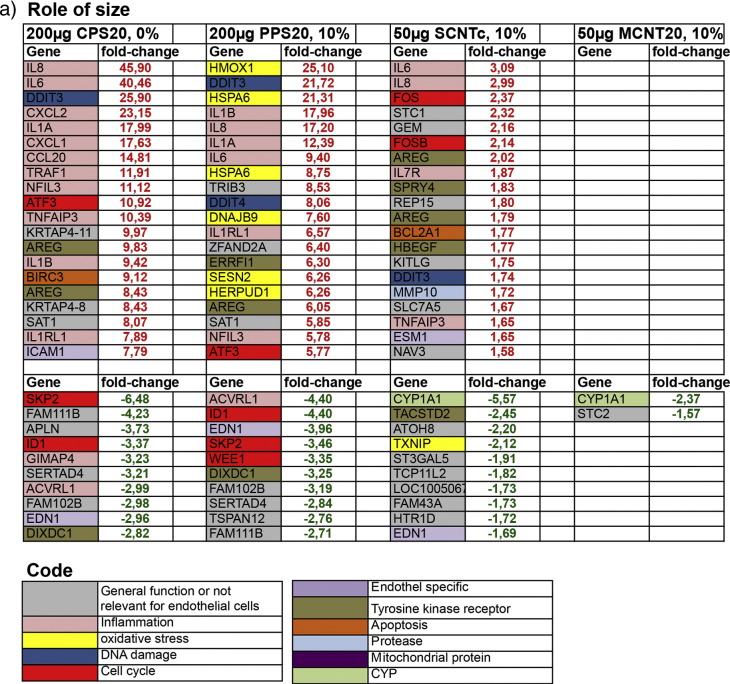

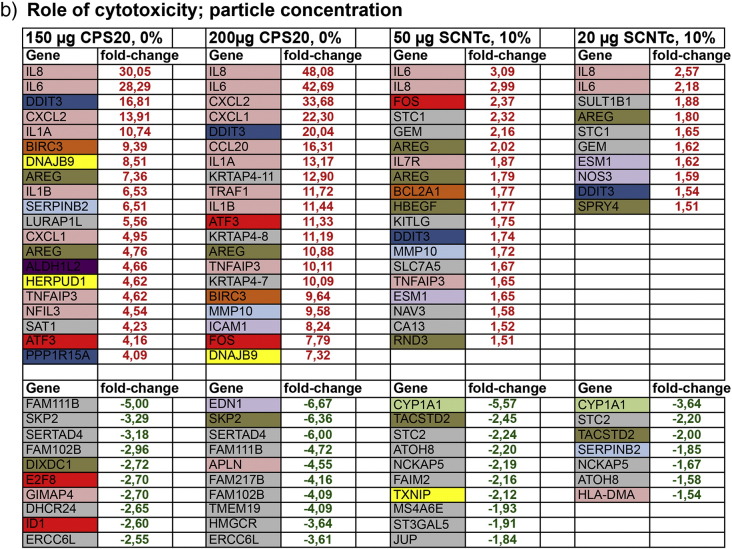

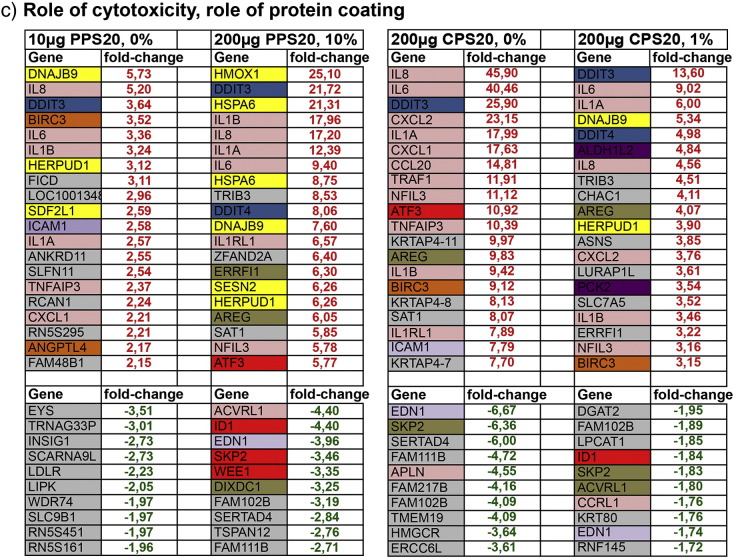
List of the 20 top up-regulated genes (fold-change in red) and the 10 top down-regulated genes (fold-change in green). Out of all whole genome expression analyses datasets to study the role of size (a), cytotoxicity; particle concentration (b), and cytotoxicity/role of protein coating (c) are listed. Genes are categorized according to GeneCards® to their localization or biological function. Headings of the columns indicate exposure conditions: particle concentration and % of FBS in DMEM.

Endothelial cell-specific genes (P- and E-selectin, VWF, VCAM-1, GAADD45B, MAPK, STAT, cyclins A and B), in general, were not regulated by the NPs used in this study. Listed among the top 20 up-regulated and the 10 top down-regulated genes, however, endothelin 1 (EDN1) was down-regulated after exposure to PPS20 (10% FBS), CPS20 (200 μg/ml) and SCNTc (50 μg/ml, Fig. 3a).

#### Genes regulated by NP exposure

Genes involved in inflammatory responses and DNA damage were up-regulated by PPS20, CPS20 and SCNTc particles (Fig. 3a). Oxidative stress-related genes, particularly involved in endoplasmic reticulum stress, were up-regulated only by PPS20 particles. CPS20 and SCNTc particles caused up-regulation of genes related to apoptosis and to cell cycle. Up-regulation of genes coding for proteases and endothelial cell differentiation were identified only in cells exposed to SCNTc.

CPS20, PPS20 and SCNTc particles caused down-regulation of genes involved in endothelial cell differentiation and tyrosine kinase signaling. CPS20 and PPS20 particles down-regulated genes involved in cell cycle and inflammatory response and SCNTc exposure genes related to oxidative stress. Non-cytotoxic NPs (CPS200) and thick CNTs (MCNT20) caused no differential regulation of well-annotated genes.

The role of cytotoxicity on whole genome expression was assessed by comparison of different concentrations of a given particle in the same exposure solution. In this setting, CPS20 particles showed concordant increases in genes involved in inflammation, DNA damage, cell cycle, apoptosis, and tyrosine kinase-signaling, and oxidative stress, while representatives of proteases and mitochondrial metabolism were up-regulated only after exposure to 150 μg/ml CPS20 particles (Fig. 3b). Up-regulation of interleukins 6 and 8 was less pronounced after exposure to 150 μg/ml than after exposure to 200 μg/ml CPS20 particles. Cells exposed to SCNTc at higher and lower dose up-regulated genes of inflammatory response, DNA damage and endothelial cell differentiation, while cell cycle related and anti-apoptotic genes were up-regulated and oxidative stress related and mitochondrial genes were down-regulated only for the higher dose. In contrast to 20 μg/ml SCNTc, higher doses of SCNTc down-regulated one oxidative stress related gene.

Since protein in the incubation solution decreased cytotoxicity for polystyrene particles, comparisons of datasets from CPS20 and PPS20 particles in DMEM with different FBS content can provide information on both the role of cytotoxicity and on protein coating. In exposures with different FBS content top-regulated genes belonged to the same functions but the number of representatives of this function was decreased. Similar to DMEM without FBS, genes involved in immune functions, in DNA damage, tyrosine kinase signaling, and in apoptosis were up-regulated by exposures to CPS20 particles in DMEM and DMEM + 1% FBS. CPS20 particles in DMEM + 1% FBS, in addition, up-regulated oxidative stress and mitochondria related genes and down-regulated one cell cycle gene, while CPS20 particles in DMEM without FBS up-regulated one cell cycle gene and down-regulated one endothelial cell-related gene (Fig. 3c). At the highest FBS concentration of 10% no annotated genes were regulated by exposure to CPS20 particles. Also 200 μg/ml PPS20 suspended in DMEM + 10% FBS reacted similarly to 10 µg/ml PPS20 particles suspended in DMEM without FBS regarding the majority of top regulated genes. Both exposures led to up-regulation of interleukin genes, oxidative stress, and DNA damage related genes (Fig. 3c). Differences were only seen in one anti-apoptotic gene and one gene for endothelial differentiation, which were up-regulated by PPS20 particles in DMEM without FBS and regarding up-regulation of tyrosine kinase signaling genes and down-regulation of cell cycle genes induced by PPS20 particles in DMEM + 10% FBS.

The role of surface functionalization could only be studied in exposures to CPS20 and PPS20 particles. Most prominent differences included higher regulation of immune system related genes by CPS20 and higher regulation of genes involved in oxidative stress response by PPS20 particles (Fig. 3a). Due to the low number of regulated genes, no conclusion on the role of carboxylation in the different CNTs was possible (Fig. 2s, Supplementary Material). All CNTs induced up-regulation of IL-8. Relatively greatest effects were seen for SCNTc, which were also the most cytotoxic CNTs. No up-regulation of genes was seen for MCNT20, MCNT20c, and MCNT50. All CNTs caused down-regulation of CYP1A1, while Serpin B2 down-regulation was only seen for the small CNTs (SCNT, SCNTc, MCNT8, MCNT8c and MCNT20c) particles.

#### Cell functions regulated by NP exposure

The greatest number of regulated genes belonged to the categories of cell death and survival, cell growth and proliferation, cell-to-cell interaction and hematological system development.

Size-dependent regulation of cancer related genes (up and down) was highest after by exposure to PPS20 particles (Fig. 3s; a, Supplementary Material). These particles down-regulated genes involved in cell death and survival, cell growth and cell cycle pathways to a greater extent than CPS particles of the same nominal size. By contrast, cell to cell interaction, hematological system development, inflammatory response, tissue development and tissue morphology were up-regulated to a greater extent by CPS20 than by PPS20 particles. CPS200 particles up-regulated cardiovascular system development, cell death and survival, hematological system development, inflammatory response, and tissue morphology pathways to a greater extent than PPS20 particles. The number of pathways regulated by SCNTc was very low. Relatively greatest effects (up-regulation) were seen for cell movement and cancer. All particles applied in DMEM without FBS induced genes for hematological system development.

CPS20 particles in the same medium (150 μg/ml vs. 200 μg/ml), in general, caused regulation of a lower number of genes at the lower than at the higher dose (Fig. 3s; b, Supplementary Material). Only for cancer and cell growth the lower CPS20 dose caused higher up-regulation of ~ 150 genes, while by the higher dose less than 100 genes were up-regulated.

CPS20 particles in DMEM with different FBS content showed that, in general, effects of CPS20 particles were mitigated in the presence of 1% FBS (Fig. 3s; c, Supplementary Material). While the most regulated pathways were still detected in the presence of 1% FBS, regulation of inflammatory response was barely detectible. Numbers of regulated genes after exposure to CPS20 particles in 10% FBS were too low for analysis by IPA.

PPS20 particles applied in DMEM + 10% FBS regulated a higher number of genes than in DMEM without FBS. These particles in the presence of 10% FBS caused greater effects on cancer-related genes and on cell development (up- and down-regulation) (Fig. 3s; d, Supplementary Material). Cell cycle, cell death and survival and cell growth were down-regulated. On the other hand, cell to cell interaction, hematological system development, cell growth, cell movement and tissue morphology were up-regulated to a greater extent when PPS20 particles were applied in DMEM without FBS.

Comparison of surface functionalization was only possible for polystyrene particles (CPS20 vs. PPS20) and results were mentioned in the first section on size. No evaluation of the role of carboxyl groups in CNTs was possible because the number of regulated genes in all CNT exposure, except SCNTc, was too low.

Effects on apoptosis, as often-reported target of NPs (e.g. Shvedova et al., 2012), are reported in section apoptosis.

### Validation by cellular assays: Interleukin secretion

**Fig. 4:**
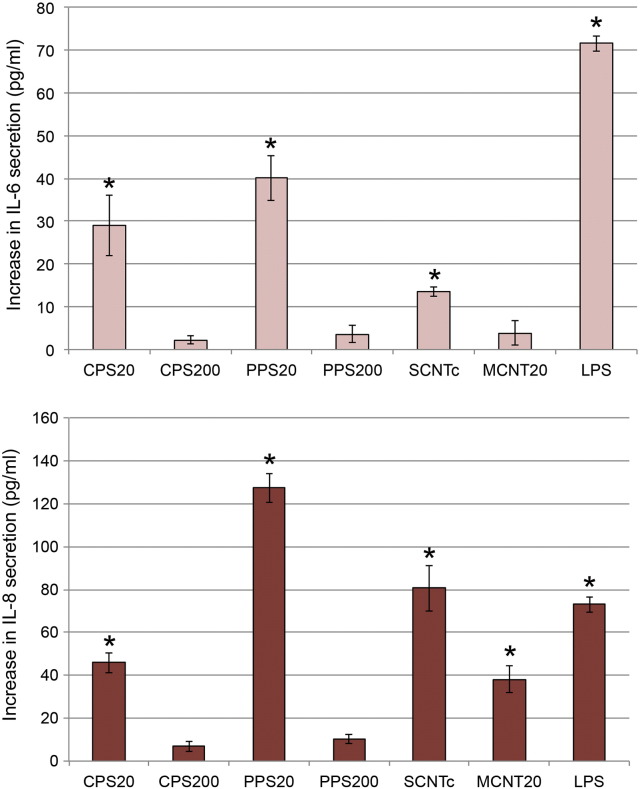
IL-6 (a) and IL-8 (b) secretion of EAhy926 cells determined after 6 h of exposure to 200 μg/ml PPS (20 nm and 200 nm), 50 μg/ml CNTs (SCNTc and MCNT20), and lipopolysaccharide (LPS) applied in DMEM + 10% FBS and 150 μg/ml CPS particles (20 nm and 200 nm) applied in DMEM + 0% FBS. Basal secretions of the cytokines in the respective medium were subtracted.

Direct comparison between gene regulation and protein expression was made for IL-6 and IL-8. Significant increases in IL-6 secretion were seen for PPS20 particles (microarray: 9.4 fold-change) followed by CPS20 particles (microarray: 28.3 fold-change) and 50 μg/ml SCNTc (3.1 fold-change; Fig. 4). IL-8 secretion was increased for PPS20 particles (microarray: 17.2 fold-change), followed by 50 μg/ml SCNTc (microarray: 3 fold-change), CPS20 particles (microarray: 30.1 fold-change) and 50 μg/ml MCNT20 (microarray: no significant change).

After exposure for 24 h IL-6 and IL-8 secretions induced by SCNTc (microarray IL-6: 2.2 fold-change; IL-8: 2.6 fold-change), MCNT8 (microarray IL-6: 1.6 fold-change; IL-8: 2.4 fold-change), and MCNT8c (microarray IL-6: no significant change; IL-8: 1.7 fold-change) were significantly higher than secretion induced by the other CNTs (Fig. 4s, Supplementary Material). In the case of IL-8 also secretion induced by MCNT50c (microarray: 1.9 fold-change) and by MCNT20c (microarray: no significant change) was significantly higher than induced by the other CNTs.

### Validation by cellular assays: Oxidative stress

**Fig. 5:**
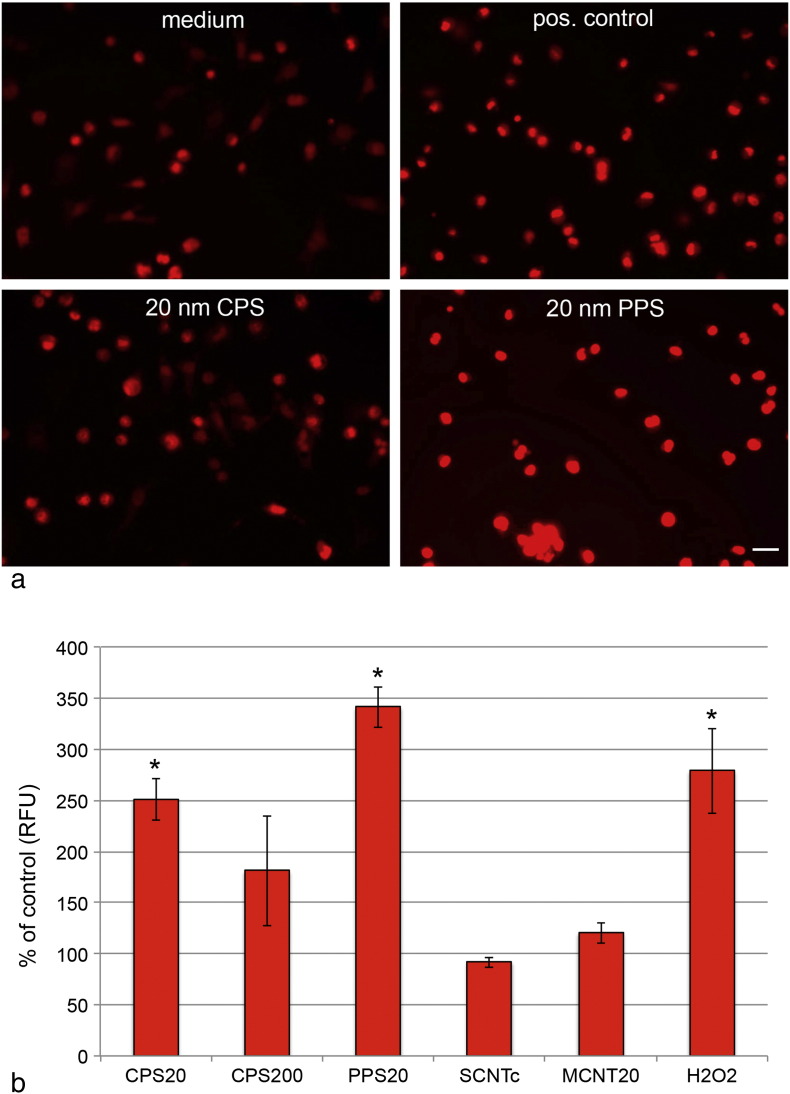
Increase of fluorescence by oxidized dihydroethidium (DHE) in EAhy926 cells exposed to positive control and to different particles. Exposure times were 24 h to 100 μg/ml CPS particles in DMEM without FBS and to 200 μg/ml PPS20 particles in DMEM + 10% FBS and 24 h to 50 μg/ml CNTs in DMEM + 10% FBS. 200 μM H_2_O_2_ in DMEM without FBS served as positive control. Data are normalized to the respective controls (DMEM with or without FBS). Significant changes are indicated by asterisk. Scale bar: 20 μm.

Microarray data indicated presence of cellular stress by activation of protective genes, such as heat shock proteins predominantly by PPS20 particles and to a lower degree by CPS20 particles. Oxidation of dihydroethidium by intracellular reactive oxygen and nitrogen species showed a similar pattern with significant increases after exposure to PPS20 and CPS20 particles. Microscopic images (Fig. 5a) confirmed the fluorometric measurements (Fig. 5b).

### Validation by cellular assays: Apoptosis

**Fig. 6:**
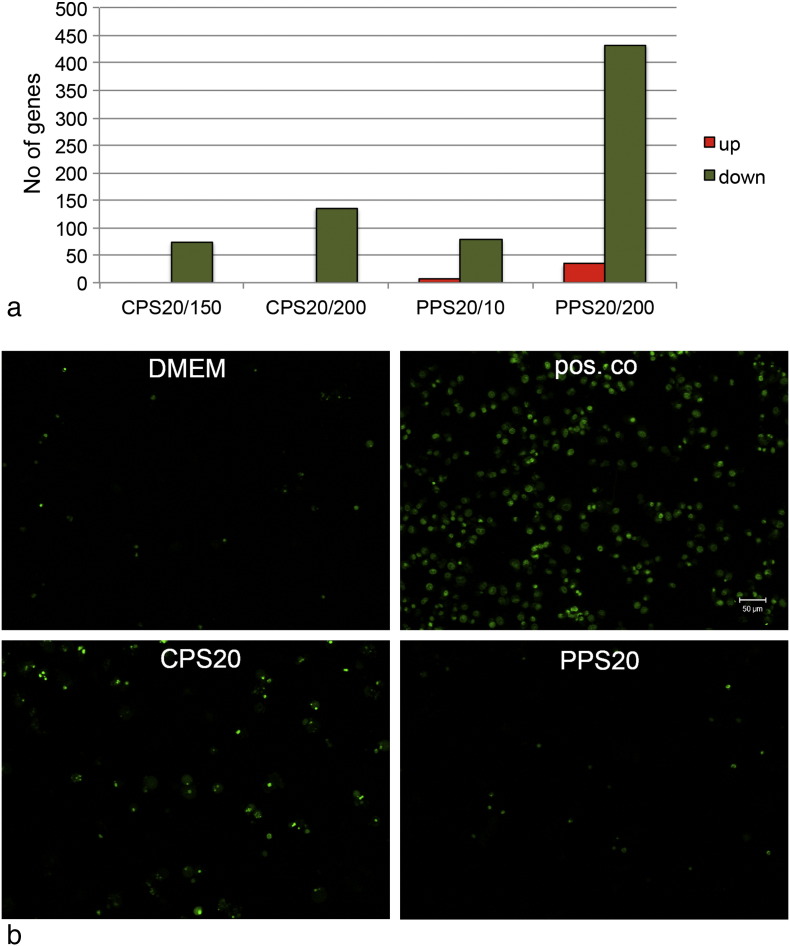
Action of particles on apoptosis. a: Number of regulated genes in the category cell death and survival, apoptosis after exposure to CPS20 particles in concentration of 150 μg/ml (CPS20/150) and 200 μg/ml (CPS20/200) and to PPS20 particles in concentrations of 10 μg/ml (PPS20/10) and 200 μg/ml (PPS20/200). b: Identification of apoptotic EAhy926 cells by YoPro-1 staining in cells exposed to medium and to particles. LPS was used as positive control in the YoPro-1 assay. Scale bar: 50 μm.

CPS20 (150 μg/ml and 200 μg/ml) and PPS20 (DMEM with and without FBS) regulated genes in the category of apoptosis (cell death and survival). In general down-regulation of genes was observed. The effect was more pronounced when higher concentrations of CPS (200 μg/ml vs. 150 μg/ml) particles were tested and when particles were applied in DMEM + 10% FBS compared to DMEM without FBS (Fig. 6a). The anti-apoptotic gene BIRC3 was up-regulated by exposure to PPS20 particles and by CPS20 particles (Fig. 3c), the anti-apoptotic genes BCL2A1 by exposure to SCNTc (Fig. 3a).

YoPro-1 staining was employed to identify apoptotic cells because many other screening assays, such as caspase 3/7 activation (Fröhlich et al., 2013), interfere with CNTs. While exposure to 200 U/ml LPS increased fluorescence significantly to 164.1 ± 18.2% of the untreated controls, there was no significant changes in the signals obtained in particle-exposed cells (Fig. 6b). Fluorescence was in the range of 87.3 and 117.7% of the untreated cells (data not shown). Fluorescence was higher after exposure to CPS20 (117.7 ± 8.2%) than to PPS20 (96.4 ± 2.9%) particles. Fluorescent images confirmed the fluorometric findings.

## Discussion

The most important finding of this study was that coating with protein had only a small effect on the pattern of regulated genes. Differences in the top regulated genes induced by polystyrene particles in more or less cytotoxic conditions (either by variation of medium composition or by varying particle concentration) were relatively small. On the contrary, both uptake and number of regulated genes were linked to the degree of cytotoxicity for all NPs.

Consistent with other findings, protein in the incubation solution decreased cytotoxicity (e.g. Fröhlich et al., 2009, Ge et al., 2011). With increasing FBS content in the medium CPS20 particles reacted less cytotoxic and the number of genes in the most regulated physiological process (inflammation, oxidative stress, and DNA damage) decreased (Fig. 3b). Cellular uptake is of key importance for cellular effects. It has been proposed that different activation of immune-related pathways by CNTs between phagocytic THP-1 cells and non-phagocytic Jurkat cells could be due to different amounts of uptake (Pescatori et al., 2013). The data of this study suggest that the smaller effect on gene regulation for CPS20 particles applied in DMEM with FBS compared to DMEM without FBS as mainly due to decreased cellular uptake. This decreased cellular uptake appears to be caused by agglomeration of particles as indicated by an increased hydrodynamic diameter of the particles analyzed by dynamic light scattering (Table 2a).

Higher cytotoxicity of 20 nm than of 200 nm polystyrene particles despite a higher % uptake rate of 200 nm than of 20 nm particles most likely is due to the fact that even at the lower uptake rate the cells contained a much higher number of 20 nm than of 200 nm particles. The same weight of CPS20 particles, for instance, contains 10^3^ times more particles than the same amount of 200 nm CPS (Prietl et al., 2014). By labeling CNTs with fluorescent BSA and following their uptake we aimed to find out whether the low number of regulated genes was due to absence of uptake of CNTs by the cells. In the comparison to uptake data for polystyrene particles it has to be taken into account that labeling with fluorescent BSA—in contrast to the labeling of polystyrene particles—is less efficient and, in addition, changed surface properties of the NPs compared to unlabeled ones suspended in FBS containing solution. Despite this limitation, this method should be suitable to identify prominent differences in particle uptake and allows the conclusion that lack of cellular contact with CNTs cannot explain the low degree of gene regulation.

PPS20 particles, which caused higher cytotoxicity in DMEM without FBS than CPS20 particles, induced differential regulation of a higher number of genes. In contrast to what has been suggested for particles with different cytotoxicity (Tilton et al., 2014), however, a rather similar gene expression profile was seen, when the top-regulated genes were compared. The only difference was activation of oxidative stress genes by exposure to PPS20 not to CPS20 particles.

The role of protein by comparison of cytotoxic concentrations of PPS20 particles in DMEM with and without FBS showed a very similar pattern when the functions of the top-regulated genes were compared. It has also been reported that depending on the protein concentration in the solution the composition of the protein coating varies and that even at very low protein concentrations the surface of NPs is completely covered by proteins (Ghavami et al., 2013, Monopoli et al., 2011). Therefore, a similar gene regulation pattern induced by PPS20 particles in the different media might indicate that the composition of the protein corona does not exert a great effect on cellular response. By comparison of gene categories/functions in IPA some differences between the exposures were seen (Figs. 3s;d). This could be due to the underlying different physiological situation of the cells in DMEM with 10% FBS and without FBS. After exposure of cells in DMEM + 10% FBS for 24 h without particles 950 genes were regulated compared to culture in DMEM without FBS (409 up-regulated and 541 down-regulated, data not shown). Only 50 genes were regulated in untreated cells cultured in DMEM without FBS compared to DMEM + 1% FBS (24 up-regulated and 26 down-regulated, data not shown). In line with this assumption, differences between CPS20 particles in DMEM without FBS and DMEM + 1% FBS based on IPA were minimal. Identification of regulated processes based on the role of the top-regulated genes appears more reliable than changes in functions based on IPA. Firstly because the analysis by IPA needs a minimum number of genes and, therefore, the included genes were only filtered by fold-changes and p-value, and secondly because genes are involved in several functions. Interleukin 6, for instance, in addition to its main function in immunological processes, influences growth and differentiation and plays a role in cancer. In the analysis by IPA there is no prioritization of these functions. Less regulated genes, which had to be included to reach the minimum of genes required for IPA, can markedly influence the obtained pattern.

While the gene regulation pattern induced by protein and protein-uncoated NPs was roughly similar, the regulation pattern for the different polystyrene particles and CNTs was particle-specific. CPS20 and CPS200 particles induced regulation of tissue morphology, hematological system development and inflammation more than PPS20 particles. On the other hand, up-regulation of cancer genes was more prominent for PPS20 than for CPS20 particles. On the basis of regulated genes, immune processes were activated more in CPS20 than in PPS20 particles treated cells, while genes related to oxidative stress were more up-regulated in PPS20 exposed cells. Up-regulation of immune processes is a common action of NPs. SiO_2_ and TiO_2_ NPs induce deregulation of inflammatory pathways after parenteral, oral and intratracheal application (TiO_2_: Chen et al., 2006, Cui et al., 2012, Gui et al., 2013; SiO_2_: Lu et al., 2013, Park et al., 2011). In the current study pro-inflammatory effects were observed in the presence (for PPS20) and absence (CPS20, SCNTs) of oxidative stress. This could be due to the fact that endothelial cells react with release of IL-6 and IL-8 not only to infections but also to other stimuli such as ionizing radiation and activated protein C (Hooper et al., 1998, Meeren et al., 1997).

The short CNTs used in this study were taken up by the cells but displayed only a low degree of cytotoxicity. Compared to another study with short SCNTs, the number of regulated genes was also very low (Alazzam et al., 2010). Since CNTs have been reported to cause gene regulation both in cytotoxic and non-cytotoxic concentrations (Chou et al., 2008, Pescatori et al., 2013, Snyder-Talkington et al., 2013), absence of cytotoxicity is not a likely reason for the low gene regulation of these CNTs. Pathways affected by CNTs in other studies included inflammation, virus response, apoptosis, oxidative stress, fatty acid metabolism and protein metabolism. CNTs in this study showed only small effects, mainly on inflammatory processes (interleukin genes), while effects on oxidative stress and apoptosis were absent in expression analysis as well as in cellular assays. Anti-apoptotic effects and up-regulated DNA repair mechanisms reported for MCNTs at low cytotoxic concentrations (Tilton et al., 2014) were also not seen in this study. This suggests a low interference of CNTs with cellular functions. In addition, little differences in the expression profiles and biological effects between the different CNTs in this study were seen. All CNTs caused down-regulation of CYP1A1 in EAhy926 cells. The relevance of this finding, when the gene is not induced, is not clear. Spot intensity of CYP1A1 on the microarray in non-treated cells was 6.8 and, according to the information of the assay producer, can be interpreted as intermediate expression. Down-regulation of CYP1A1 has also been shown in cells of the respiratory tract, breast cancer cells and hepatocytes exposed to SCNTs (Hitoshi et al., 2012). In endothelial cells CYP1A1 is constitutively expressed and participation in inactivation of circulating protoxicants has been proposed (Farin et al., 1994). A main regulator of CYP1A1 in endothelial cells is shear stress (Conway et al., 2009), suggesting an additional role in other endothelial processes.

Only interleukins IL-6 and IL-8 could be compared on the mRNA basis and on protein detection. The degree of protein expression by PPS20, SCNTc and MCNT20 was similar to the fold-changes in the microarray analysis. Protein levels induced by CPS20 particles were lower than estimated from whole genome expression data. The reason for these reduced levels might be a lower rate of protein synthesis in serum-deprived cultures (Larsson et al., 1985) combined with a lower degree of protein synthesis upon stimulation in cells cultured in serum-free medium (Grieninger et al., 1978).

The data suggest that cell-based assays for inflammation, cell cycle, apoptosis and oxidative stress represent the changes observed in whole genome expression quite well. Protein in the incubation solution, leading to formation of a protein corona and causing aggregation, decreased cytotoxicity. Similar processes were regulated by the NPs but the number of regulated genes belonging to these processes was decreased.

## Conflict of interest

The authors declare that no competing financial interests exist.

## Acknowledgment

This work was supported by the 10.13039/501100002428Austrian Science Fund grant N214-NAN and P 22576-B18.
