# Supplementary material for: Use of whole genome expression analysis in the toxicity screening of nanoparticles
Source: Toxicol Appl Pharmacol. 2014 Oct 15;280(2):272–84. doi: 10.1016/j.taap.2014.07.017 (PMC4222661; doi:10.1016/j.taap.2014.07.017)
Supplement: Supplementary file 1 — Supplementary figures [file mmc1.docx]

# Supplementary Material

#
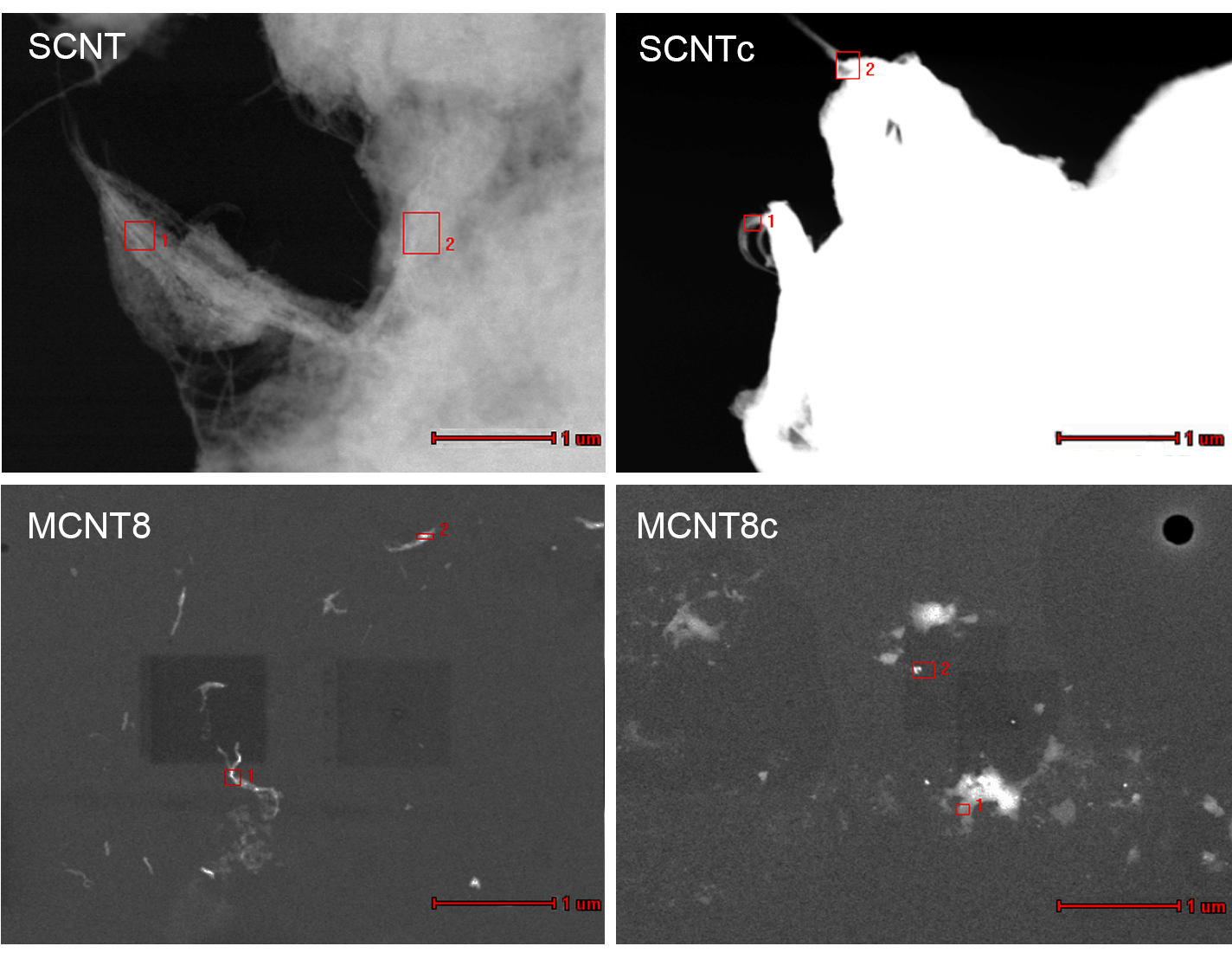


#
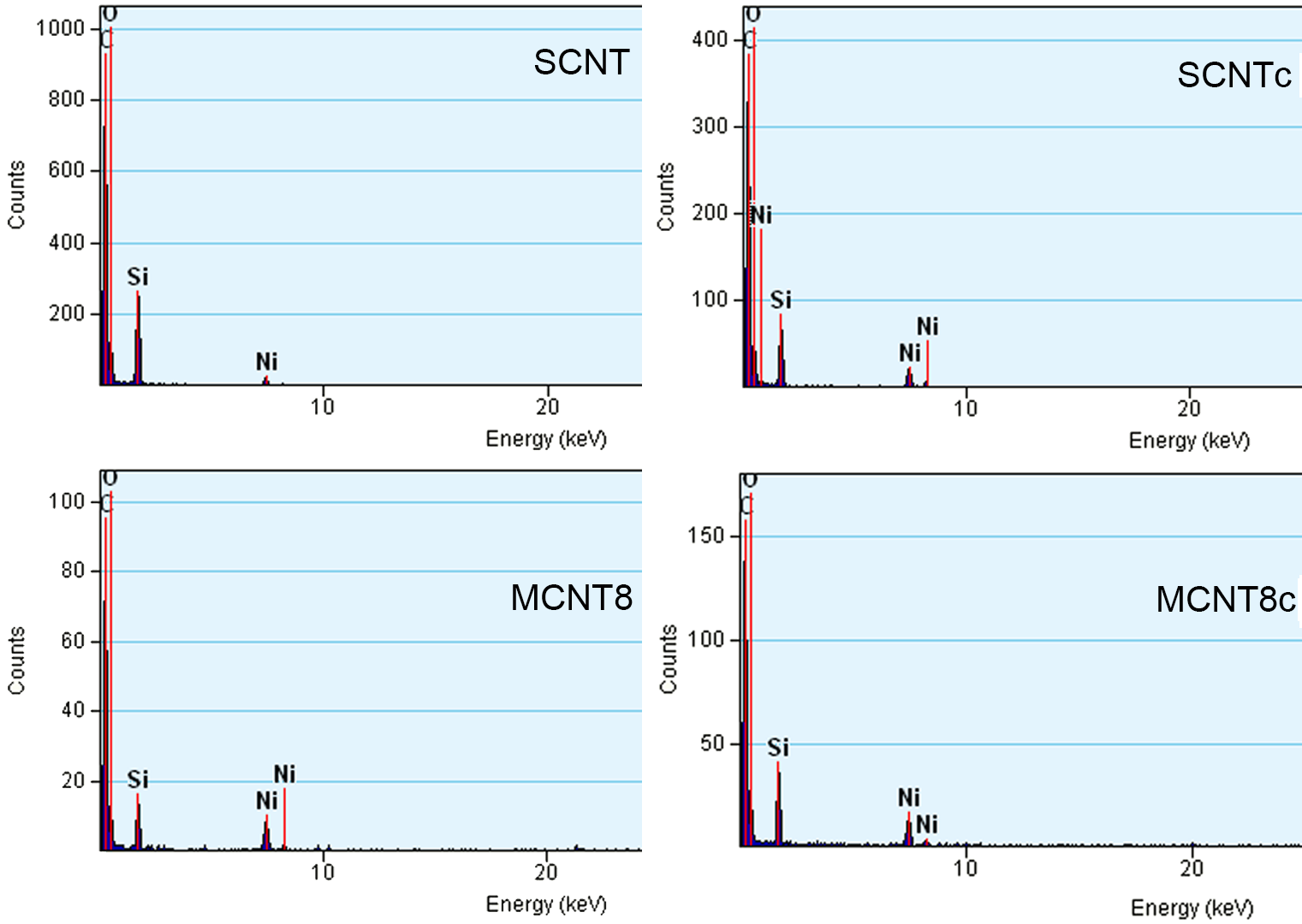


Fig. 1s: Dark-field scanning TEM images of CNTs dispersed in water with indication of areas used for x-ray energy dispersive spectrum (EDS) analysis (upper panel). Measurements were made in areas where tubes were singular. In the lower panel EDS spectra of representative areas of functionalized (SCNTc, MCNT8c) and pristine CNTs (SCNT, MCNT8) are seen.


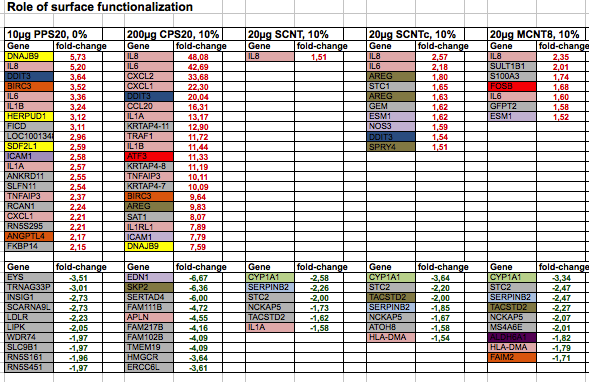


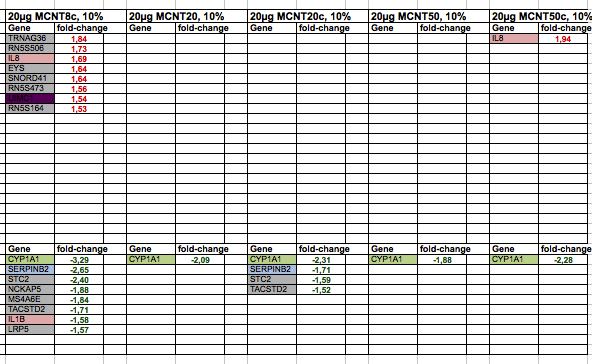


Fig. 2s: Top differentially regulated genes for particles with different surface functionalization of polystyrene particles (tested in DMEM + 0% FBS) and CNTs (tested in DMEM + 10% FBS). Changes of up-regulation compared to the respective cultures without particles are indicated in red and changes of down-regulation in green. 0%: DMEM + 0%FBS; 10%: DMEM + 10%FBS.


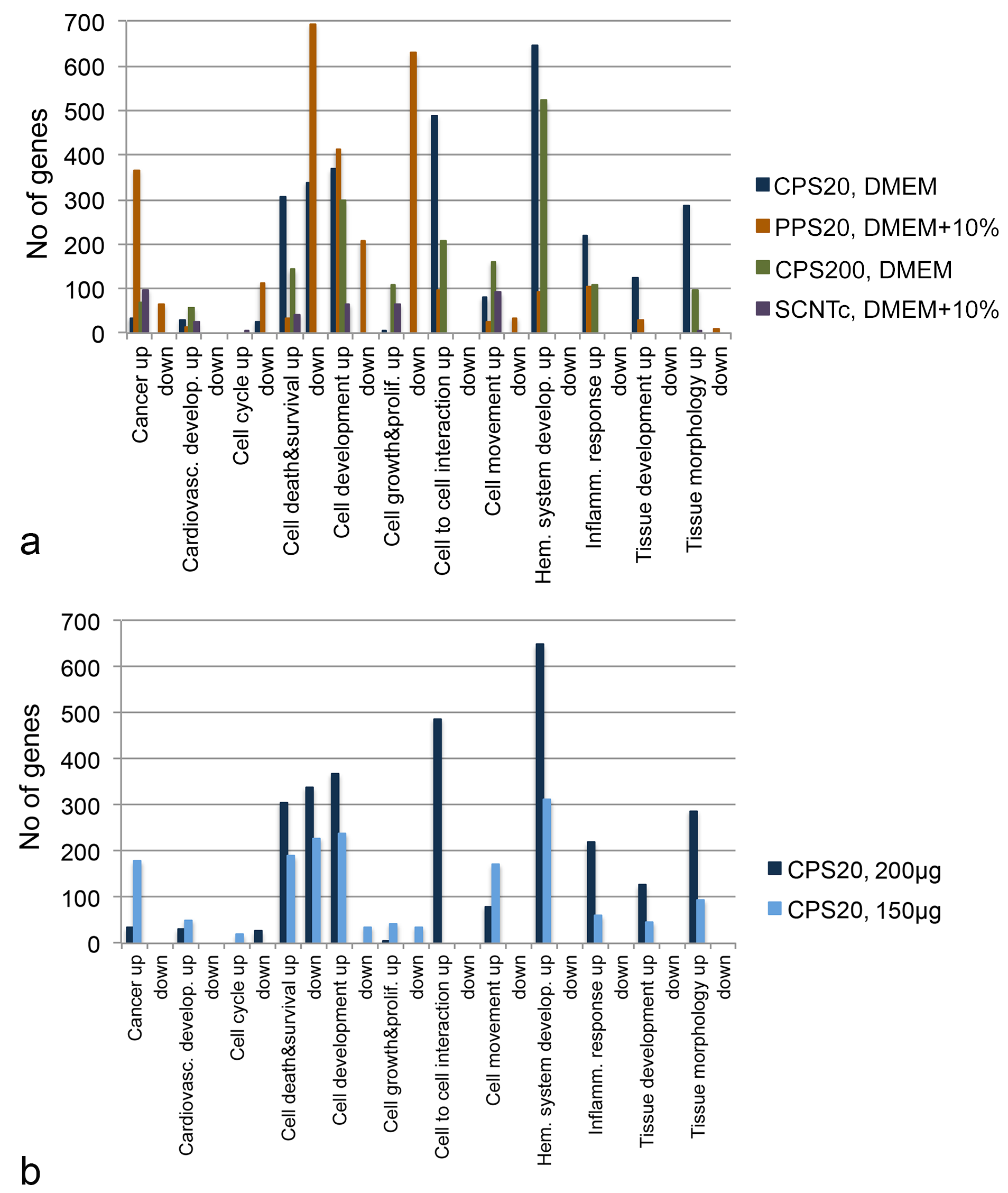


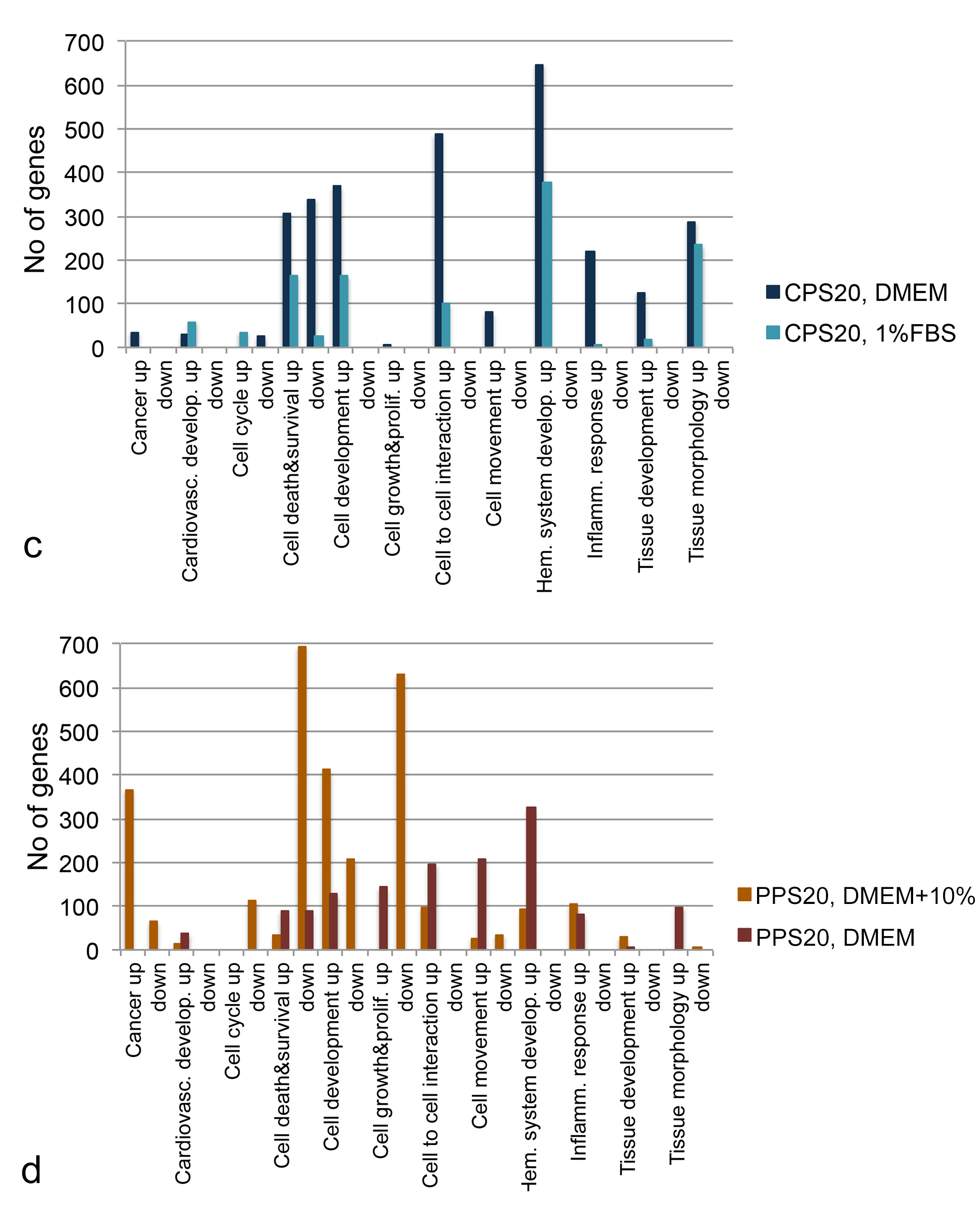


Fig. 3s: Comparisons of differentially regulated gene functions (Ingenuity) regarding particle size (small and large polystyrene particles and thin and thick CNTs, a) cytotoxicity (different concentrations of particles in the same medium causing different extent of cytotoxicity, b), cytotoxicity and protein coating (particles applied in different media causing different extent of cytotoxicity, c and particles applied in different media causing the same degree of cytotoxicity, d).

Abbreviations: CPS20, DMEM: 200µg/ml in DMEM without FBS; PPS20, DMEM+ 10%: 200 µg/ml PPS20 particles in DMEM + 10% FBS; CPS200, DMEM: 200 µg/ml CPS200 particles in DMEM without FBS; SCNTc, DMEM+10%: 50 µg/ml SCNTc in DMEM + 10% FBS; CPS20, 200µg: 200µg/ml in DMEM without FBS; CPS20, 150µg: 150 µg/ml CPS20 in DMEM without FBS; CPS20, DMEM: 200 µg/ml CPS20 in DMEM without FBS; CPS20, 1% FBS 200µg/ml CPS20 in DMEM + 1% FBS; PPS, DMEM+10%: 200 µg/ml in DMEM + 10% FBS; PPS20, DMEM: 10 µg/ml PPS20 in DMEM without FBS


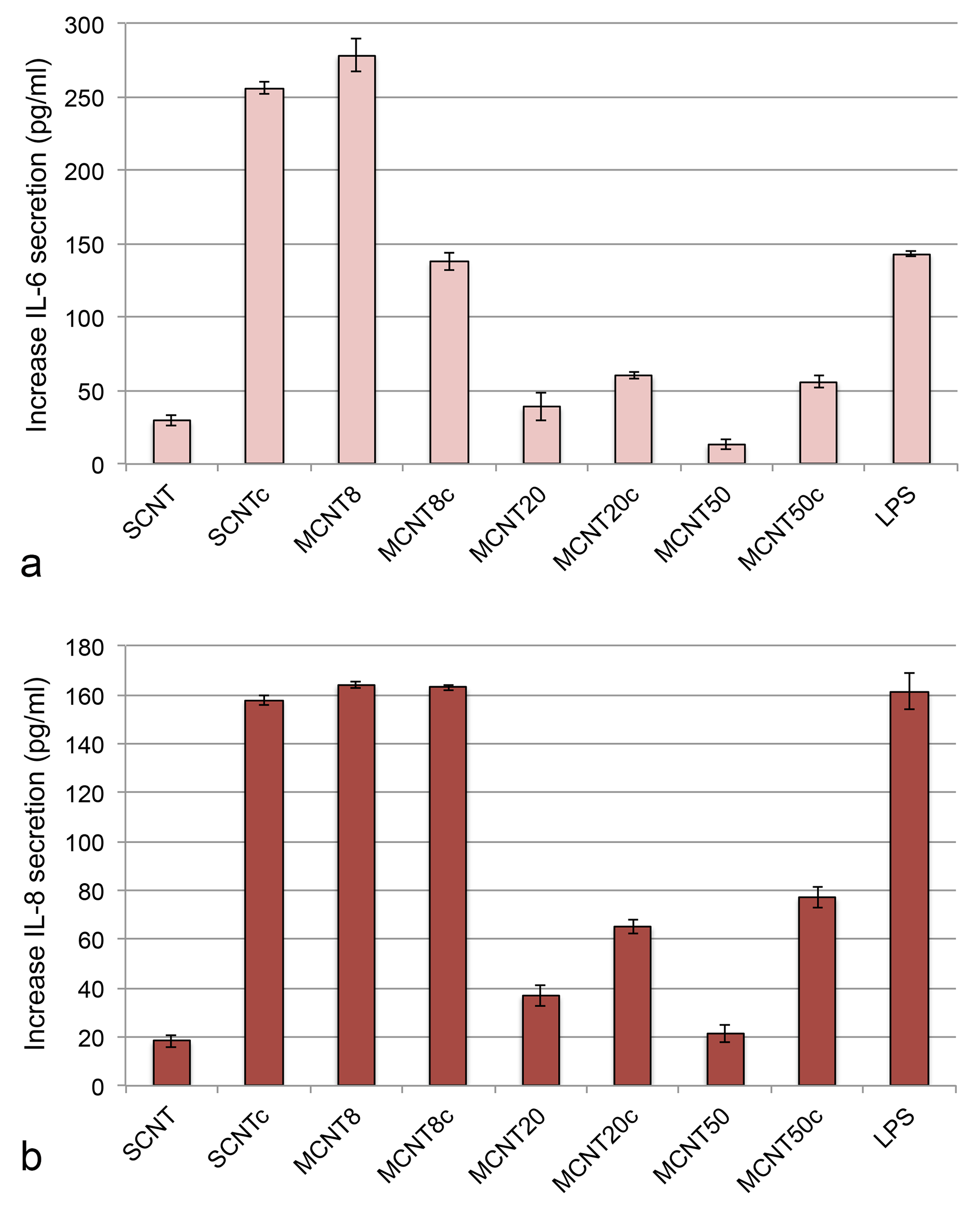


Fig. 4s: Interleukin secretion after exposure of EAhy926 cells to 20 µg/ml CNT in DMEM+10% FBS and to LPS as positive control for interleukin secretion for 24h. All increases are significant with the exception of IL-6 secretion by MCNT50.
